# An exploration of group-based HIV/AIDS treatment and care models in Sub-Saharan Africa using a realist evaluation (Intervention-Context-Actor-Mechanism-Outcome) heuristic tool: a systematic review

**DOI:** 10.1186/s13012-017-0638-0

**Published:** 2017-08-25

**Authors:** Ferdinand C. Mukumbang, Sara Van Belle, Bruno Marchal, Brian van Wyk

**Affiliations:** 1 School of Public Health, University of the Western Cape, Cape Town, South Africa; 2 Department of Public Health, Institute of Tropical Medicine, Antwerp, Belgium

**Keywords:** Adherence, Group-based ART model, Antiretroviral therapy, Narrative synthesis, Retention in care, Realist evaluation, Theory-driven review

## Abstract

**Introduction:**

It is increasingly acknowledged that differentiated care models hold potential to manage large volumes of patients on antiretroviral therapy (ART). Various group-based models of ART service delivery aimed at decongesting local health facilities, encouraging patient retention in care, and enhancing adherence to medication have been implemented across sub-Saharan Africa. Evidence from the literature suggests that these models of ART service delivery are more effective than corresponding facility-based care and superior to individual-based models. Nevertheless, there is little understanding of how these care models work to achieve their intended outcomes. The aim of this study was to review the theories explicating *how* and *why* group-based ART models work using a realist evaluation framework.

**Methods:**

A systematic review of the literature on group-based ART support models in sub-Saharan Africa was conducted. We searched the Google Scholar and PubMed databases and supplemented these with a reference chase of the identified articles. We applied a theory-driven approach—narrative synthesis—to synthesise the data. Data were analysed using the thematic content analysis method and synthesised according to aspects of the Intervention-Context-Actor-Mechanism-Outcome heuristic-analytic tool—a realist evaluation theory building tool.

**Results:**

Twelve articles reporting primary studies on group-based models of ART service delivery were included in the review. The six studies that employed a quantitative study design failed to identify aspects of the context and mechanisms that work to trigger the outcomes of group-based models. While the other four studies that applied a qualitative and the two using ﻿a mixed methods design identified some of the aspects of the context and mechanisms that could trigger the outcomes of group-based ART models, these studies did not explain the relationship(s) between the theory elements and how they interact to produce the outcome(s).

**Conclusion:**

Although we could distill various components of the Intervention-Context-Actor-Mechanism-Outcome analytic tool from different studies exploring group-based programmes, we could not, however, identify a salient programme theory based on the Intervention-Context-Actor-Mechanism-Outcome heuristic analysis. The scientific community, policy makers and programme implementers would benefit more if explanatory findings of how, why, for whom and in what circumstances programmes work are presented rather than just reporting on the outcomes of the interventions.

**Electronic supplementary material:**

The online version of this article (doi:10.1186/s13012-017-0638-0) contains supplementary material, which is available to authorized users.

## Background

Sub-Saharan Africa (SSA) remains the most severely affected region by the HIV and AIDS pandemic, accounting for nearly 71% of the people living with HIV (PLWHA) worldwide [1]. In response to the hyper-epidemics in various countries in this region, and with support from major Global Health Initiatives, HIV treatment programmes—following evidence of the effectiveness of ART—were rapidly expanded from 2005 [2]. Currently, there is evidence that HIV treatment and care can be used to foster the ‘test, treat, suppress, and prevent’ approach to controlling the HIV pandemic, an approach believed to potentially end AIDS by 2030.

Antiretroviral treatment (ART) is a ‘holistic’ treatment approach, whereby taking antiretroviral drugs in compliance with the treatment protocol, eating healthy, and receiving psychosocial support and palliative care is provided as a package [3]. Patients who follow most or all the components of ART have shown improvement in their viral load readings (< 400 copies/mL), an increase in the CD4 count (> 200 cells/mm^3^), a lower incidence of opportunistic infections, and an overall improvement in health [4]. For PLWHA to benefit from all aspects of ART, they must be retained within the care umbrella—that is patients need to be tested for HIV, initiated on treatment, retained in care and reach and maintain viral suppression. Ensuring that patients are retained in care is, therefore, crucial for reaching and maintaining viral suppression and by extension, a successful ART programme [5]. The concept of patient-focused care that has received support for clinic patient management in recent times has promoted individual-focused ART adherence. Nevertheless, it is argued by Haberer et al. that successful population-level ART adherence is pivotal to realising the clinical and prevention benefits of antiretroviral scale-up and consequently ending AIDS by 2030 [6].

As HIV treatment and care programmes mature and extend over the years, the need to ensure long-term retention in care for patients receiving ART while continuing timely initiation of new patients into treatment presents an ongoing challenge to healthcare providers and policy makers [7]. Although the mainstream treatment scheme (facility-based ART services) has gone a long way towards providing the necessary clinical care that patients on ART need to achieve and sustain viral suppression [8], increasing numbers of patients initiated on ART decreases quality care delivery, making it difficult to maintain population-level adherence. It is estimated that in SSA, on average, only 64% of patients are retained in care after 2 years on ART [9]. Such sub-optimal retention in care rates have implications for the new three-part HIV treatment labelled ‘90-90-90’ by 2020 and for the vision to end AIDS by 2030 [10].

Evidence from various studies reveals that task-shifting, where specialist clinical care is delegated to primary health care facilities, makes efficient use of resources without compromising patient outcomes [11, 12]. Nevertheless, this strategy has a limited capacity to provide long-term resolutions to the challenges of patient retention in care if clinics are overcrowded and waiting times are long, as is often the case in South Africa. Consequently, a continuum of strategies ranging from health service-driven to client-driven options has been developed and implemented to optimise ART delivery in various countries in SSA [13–15].

### Alternative antiretroviral treatment models of care

In responding to the need of scaling up treatment for millions of PLWHA while retaining those already in HIV treatment and care, various differentiated care models have been developed. Differentiated care is defined as ‘a client-centred approach that simplifies and adapts HIV services across the cascade to reflect the preferences and expectations of various groups of people living with HIV while reducing unnecessary burdens on the health system’ [16].

The standard facility-based care model usually involves patients visiting the health care facility on a monthly basis to be seen by a clinician for routine consultation, and then by a lay counsellor for their drug accountability assessment and counselling. The patient is then provided with one month’s supply of medication from the pharmacy. Differentiated care models on the other hand, usually integrate most of the services provided by the standard care model and offer it as a tailored package to the suit the needs of different types of patients (patient groups). For instance, patients can be provided with their medication, education and counselling and monitoring services when they are part of a differentiated care model. Differentiated care models are different from mainstream ART service delivery in that they streamline ART services by adapting the care components to the needs of differentiated groups of patients.

Differentiated models usually cover the core activities for providing ART while addressing some of the challenges that patients who use the mainstream clinical care face such as long waiting times, poor access to medication and long distances to the clinic. These differentiated models usually provide minimal clinical screening, quick antiretroviral medication refills, adherence support and defaulter tracing, strict monitoring of attendance and problem-solving, and encouraging self-efficacy and mutual support [17]. Therefore, differentiated models of ART service delivery usually adopt a multifaceted approach towards achieving better adherence and retention in care rates. They increase the capacity and efficiency of ART service delivery by tailoring services according to the needs of different patient groups, reducing clinic contact and relying on community-based services for those who are stable on ART.

Most differentiated care models were developed and implemented by organisations such as Médecins Sans Frontières (MSF) and the AIDS Support Organization (TASO) in collaboration with various local governments in SSA [13]. Following their implementation success in pilot projects, differentiated models are increasingly recognised as an essential approach to managing patients on ART [18]. According to Campion, for these alternative treatment models to be successful, they should ensure that drug delivery is patient-centred, they should fit into the lives of the patients, require minimal time, and they should not be linked to clinical consultations [19].

**Fig. 1:**
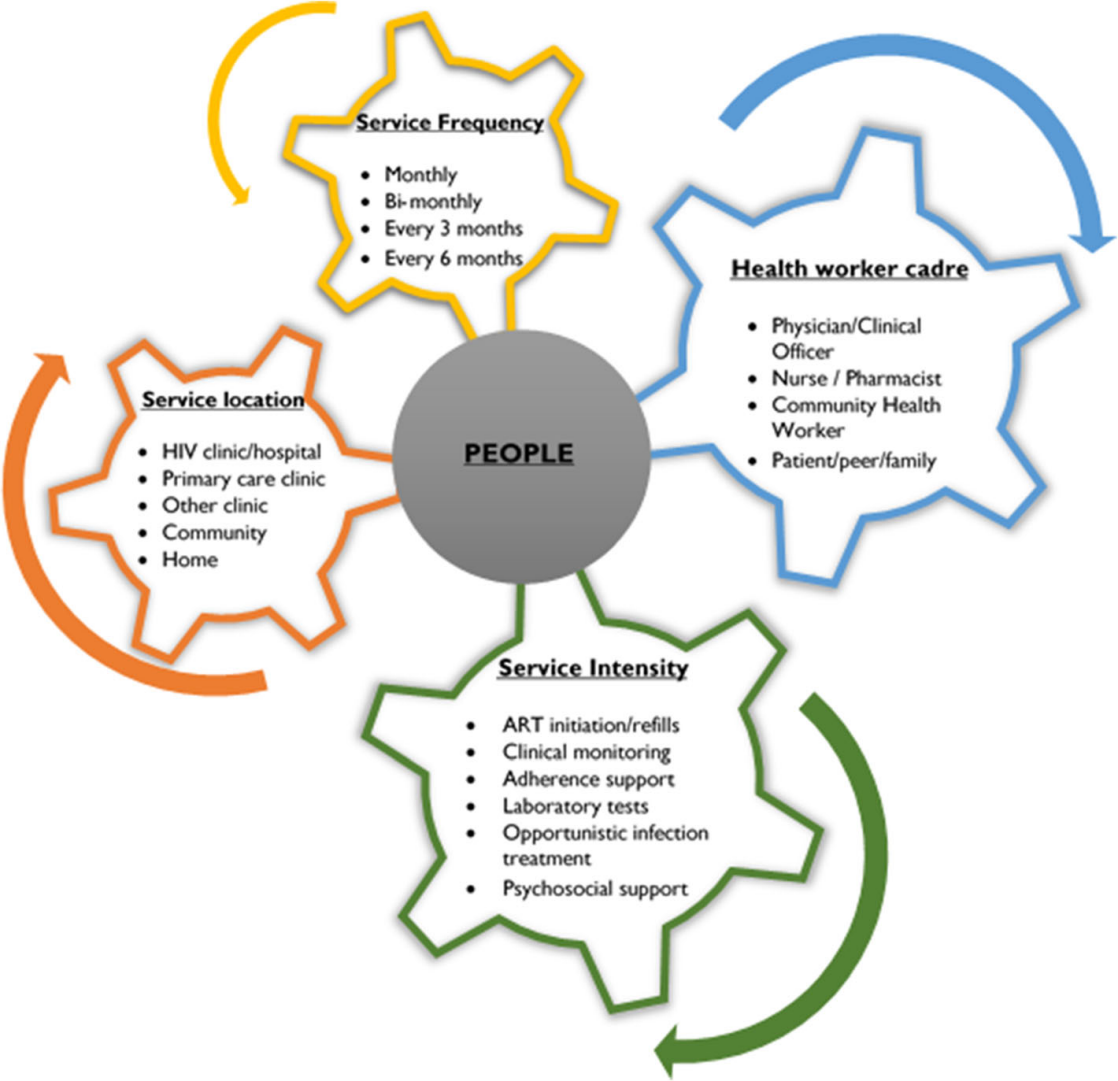
A framework for differentiated models of ART services (Adapted from Duncombe et al. [17])

The new WHO guidelines (2015) highlight the need for differentiated care frameworks with variations in service frequency, health worker cadre, service location and service intensity across countries and populations [18]. Duncombe and colleagues designed a framework for HIV treatment services which identifies variations in the intensity of the core programme components that are tailored to the specific needs of different groups of individuals across the cascade of HIV services [20]. This framework focuses on service intensity of four delivery components: type of services delivered, the location of service delivery, the cadres of health service providers involved, and frequency of visits to health [17]. Fig 1 displays this framework.

Differentiated care can either be healthcare worker-managed groups or client (patient)-managed groups and can either be facility-based individual model or out-of-facility individual model [16]. Most individual-focused models of ART service delivery focus on providing psychological support, adherence monitoring and equally address household dynamics potentially impacting on patient adherence [12, 14]. The effects of adherence-enhancing interventions targeting individuals are usually small to modest. Because individual-focused models are usually resource-intensive, they tend to fade over time [21]. While group-based care models offer psychological support and ensure easy access to medication and adherence monitoring, they also create a conducive atmosphere for peer-support among the group members. Individual-focused models such as home-based care [22, 23] and ‘accompagnateur’ (adherence supporter) [24–26] do not have this aspect of care, except in situations where two or more family members are receiving ART.

Group-based ART delivery models operate on the notion that patients on ART have a mutual self-interest to receive convenient and quality ART services in a conducive and supportive environment, free of stigma and discrimination. To improve the aspect of convenience, most group-based models of ART and HIV care are out-of-clinic care models—brought close to patients. Examples of the above mentioned include community ART distribution, community ART groups, and community–based ART adherence clubs. Table 1 below presents alternative models for the delivery of long-term ART.

**Table 1:** Summary of strategies for differentiated models for delivery of long-term ART [13, 20]

Key objective		ART adherence clubs		CADP	CAG	CHBC
		Facility-based club	Community-based club			
Patient perspective	Reduce cost (time + transport)	• Reduction of clinical visit • Less time spent at clinic for drug refill	• Reduction of clinical visit • Less time spent a clinic for drug refill	• Reduction of clinical visit • Less time spent at clinic for drug refill	• Reduction of clinical visit • Less time spent at clinic for drug refill	• Reduction of clinical visit • Less time spent at clinic for drug refill
	Increase peer support	At club in health facility and potentially beyond into community	At club in community and beyond	At distribution points by expert patients	At CAG meetings in community and beyond	At HBC meetings by the CHWs
	Enhance community participation	No	Potentially	Potentially	Potentially	Potentially
Healthcare service perspective	Reduce workload					
	• Nurse	Yes	Yes	Yes	Yes	Yes
	• Pharmacist	No	No	Yes	Yes	Yes
	• Counsellor/CHW expert patient	No (facilitation by club)	No (facilitation by club)	No (Distribution and monitoring)	No (formation, training and supervision of CAGs)	No (formation, training and supervision of HBC)
	Maintain/improve health outcomes					
	• Adherence	Yes	Yes	Unknown	Unknown	Unknown
	• Retention	Yes	Yes	Yes	Yes	Yes
	Improve self-management of patient	Adherence support	Adherence support and tracing	Organisation of service for drug refill, adherence support, tracing and testing	Drug refill, adherence support, tracing and testing	Adherence support and tracing
	Decongest facility	No	Yes	Yes	Yes	Yes

*CADP* community ART distribution point, *CAG* community ART groups, *CHBC* community home-based care

The effectiveness of group-based ART models in retaining ART patients in care and improving adherence to medication has been assessed in different studies in SSA. Studies on the community ART group (CAG) model in Tete, Mozambique, have shown better retention in care rates and lower mortality among ART patients enrolled in the CAG model compared to patients in standard facility-based clinical care [27, 28]. Studies on both the facility-based and community-based ART adherence club models in the Western Cape Province of South Africa have shown better retention in care and adherence to medication rates among patients compared to those patients in standard ART care [29, 30]. Also, based on the findings of a systematic review to assess the effectiveness of group-based adherence models of care, it was concluded that community support programmes could be an effective strategy to improve the effectiveness of ART treatment and care in SSA and elsewhere [31].

It is argued by Chen [32] that theory-driven approaches to programme development improve implementation. The theoretical understanding of how and why individual-focused interventions work has been explored previously using various health behaviour theories [33]. On the other hand, because group-based ART interventions models are complex—typically multi-component and context-dependent—they are challenging to replicate and evaluate [34]. In most instances, not even the programme theories—an explicit theory or model of how an intervention contributes to a set of specific outcomes [35]—of group-based ART interventions models are described. Therefore, our understanding of *how* and *why* group-based ART models achieve better adherence and retention in care rates within their context is limited. To this end, we aimed to review primary studies systematically and develop a narrative synthesis of the mechanism(s) at work during the implementation of group-based interventions for ART adherence support. Identifying the causal mechanisms of group-based ART models is the precursor step in developing programme theories that explain *how* and *why* group-based ART treatment and care models work, for which ART population group and under what circumstances.

There are three steps in eliciting a programme theory in realist evaluation [36]. Step 1 entails conducting an exploratory qualitative study to identify the assumptions of the programme designers and health service managers on how and why the adherence club intervention is expected to achieve its goals and perceptions on how it has done so. These assumptions are also called ‘folk theories’ [37]. In the second step, we reviewed theories on ART adherence to identify candidate/potential mechanisms provided by ART interventions [38]. The third step, which is the focus of this paper, involves assessing the evidence on how and why group-based ART adherence interventions work by examining their underlining theories using the context-mechanism-outcome (CMO) heuristic—a realist evaluation analytic tool.

## Review questions

Three research questions guided the review:

1. What are the key mechanisms that drive the outcomes of group-based ART adherence interventions?
2. What are the contextual factors that influence the triggering of mechanisms, the implementation and the outcomes of group-based ART adherence interventions?
3. Are there identifiable pathways to the outcomes? If so, how do the key mechanisms and contexts interact to produce these outcomes?

### Realist evaluation and generative mechanisms

Realist evaluation is underpinned by a ‘generative’ model of causality [38]. Identifying the ‘generative mechanism’ is at the core of eliciting the programme theory. Generative mechanisms describe the causal forces, powers, processes or interactions that generate change within an intervention—including the choices, reasoning and decisions that people make as a result of the resources provided by the programme. Therefore, the key explanatory element in realist evaluation is the generative mechanism, which elucidates the reasoning the actors attribute to the resources, opportunities and/or restraints provided by the intervention that leads to action. A generative mechanism in realist logic is thus mathematically represented as:

Resources (constraints and/or opportunity) + Reasoning = Mechanism [39].

**Fig. 2:**
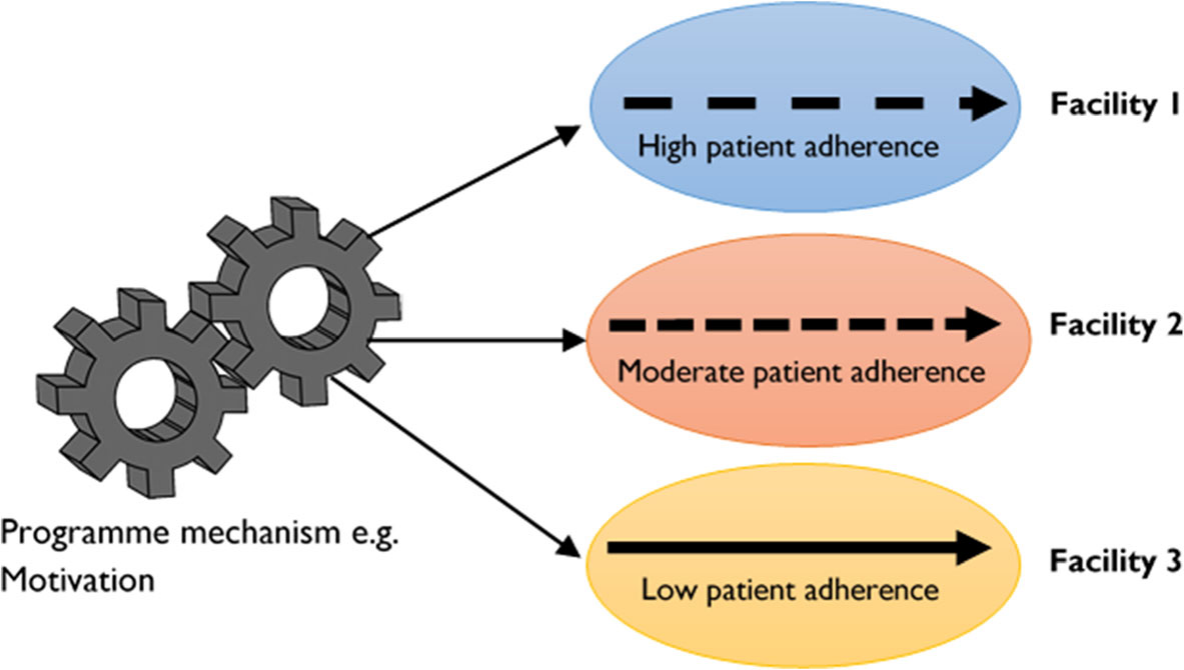
The same mechanism is postulated as generating contrasting outcomes

The same mechanism can produce different results in different contexts (Fig 2). This suggests that while the same intervention might instigate the same mechanism(s), the differences in the outcome(s) of an intervention in different settings could be largely associated with the differences in the context within which the intervention is implemented. For instance, if ‘motivation’ (mechanism) is stimulated by adherence counselling and educational talks (intervention), to improve ART adherence (outcome) among PLWHA (actors), then possible context variations could include the pre-existing group dynamics within the adherence club, the local socio-economic conditions, the local cultural norms, the geographical accessibility of the club, etc. The educational level of patients, the meaning they attach to their disease, and the way they cope with it are examples of other patient-related factors that are likely to vary substantially. The level of adherence to medication and retention in care in the different contexts represented by different health care facilities depends on how much the motivation is triggered by the intervention and modified by the various context conditions.

Formulating an initial programme theory in realist evaluation involves adopting a ‘generative’ approach to causation. Pawson and Tilley [37] identified context, mechanisms and outcomes as the three tenets to explaining how and why programmes work or not with the notion that an outcome (O) is generated by a mechanism (M) being triggered in context (C). Considering that an intervention works through actors, generative *mechanisms* (M) are used to explain how the *intervention* (I) (or aspects of the intervention) unfolds in a particular *context* (C) and in relation to the various *actors* (A) to produce the observed *outcomes* (O). Representing the intervention modalities (I) and the relevant actors (A) provides a comprehensive representation of how, why, for whom and under what circumstances a programme works (or not) [40]. Following this causality conceptualisation, we considered an intervention-context-actor-mechanism-outcome (ICAMO) configuration to provide a comprehensive analytical tool to account for (or explain) aspects of the intervention that provide the mechanisms and the actors through whom the intervention works. In this article, we adopted the ICAMO heuristic tool for the analysis purpose.

To explore the potential generative mechanisms that group-based ART models could provide through their various modalities, we carried out a narrative synthesis of the literature, a theory-based review approach, which allows us to explore components of the intervention implementation to identify their underlining theory or theories. This study was conducted as part of larger research project ‘A realist evaluation of the antiretroviral treatment adherence club programme in selected primary health care facilities in the metropolitan area of Western Cape Province, South Africa’ [41]. The first phase of this project involves eliciting the initial programme theory of the adherence club intervention—what aspects of antiretroviral club intervention work, for what sections of the patient population, and under which community and health systems contexts. An important part of eliciting the initial programme theory is reviewing the literature on how similar interventions that have been implemented are postulated to work, why they work, for whom they work and under what circumstances [42], and this paper presents the findings of that step.

## Methods

### Study design

We used narrative synthesis (NS)—an approach used to systematically review and synthesise findings from multiple studies. It relies primarily on the use of narratives to summarise and explain the findings of a synthesis [43]. NS is applied when statistical meta-analysis (for quantitative studies) or meta-ethnography (for qualitative analysis) is not possible and when the existing literature includes a wide range of interventions and studies with different (study) designs, which because of the heterogeneity cannot be pooled for analysis [43, 44]. Pawson and colleagues [45] suggested that theory-driven reviews are more fruitful approaches to reviewing evidence when evaluating competing interventions addressing the same problem.

NS fits with the logic of realist evaluation—NS requires the reviewer(s) to develop a theory of how the intervention in question works, why and for whom [43]. NS is recommended for reviews addressing questions on the effects of interventions, particularly, the implementation of interventions that are proven to be effective in experimental settings [46]. NS suits the study because most group-based ART interventions have been shown to be effective in pilot studies, a recommendation for its use [43, 46]. Finally, the heterogeneity of the literature on group-based ART interventions warrants the use of narrative reviews.

NS also fits in with the formulation of the initial programme theory as conceived by realists. The guidance on NS in systematic reviews focuses on the effects of interventions (‘outcomes’) and the factors influencing intervention implementation (which aligns with the notions of ‘mechanism’ and ‘context’). This approach is thus well-matched with the realist logic. According to Arai and colleagues [46], the ‘theory of change’ identified in the first step of a NS provides a way for abstracting the mechanisms into propositions at the end of the synthesis process. Finally, NS is aligned with the epistemological position of realist evaluation.

### Identification and selection of studies

The search process proceeded in two phases. First, we conducted an exploratory search to enable us to make an initial judgement on the availability of evidence to answer the review questions. Second, after confirming that there is adequate literature, we searched for primary research studies for possible inclusion in the review. We applied two methods to search for literature: electronic database searching (PubMed and Google Scholar) using keywords and snowballing of citations from the reference list of other authors. The goal was to find primary studies that shed light on the explanatory model of how the group-based ART models work.

**Fig. 3:**
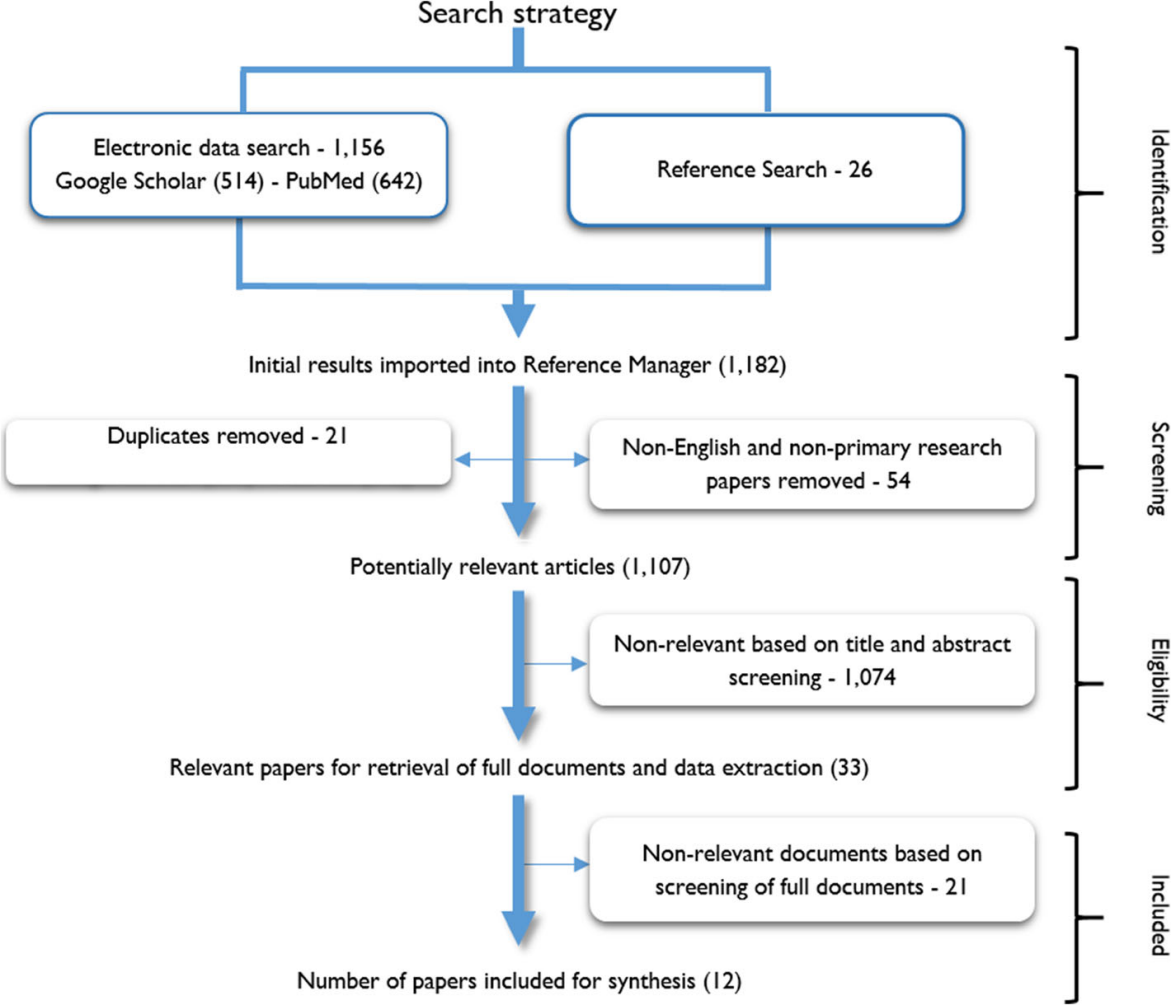
Article screening process based on the PRISMA protocol

The following search MeSH terms were used for the search of the electronic databases: ‘community-based antiretroviral therapy programme’, ‘group-based antiretroviral therapy’, ‘Interventions to improve adherence to antiretroviral therapy and retention in care’, ‘Implementation of community-based antiretroviral therapy programmes’, ‘Effectiveness of antiretroviral treatment and care interventions’, ‘Differentiated antiretroviral treatment models’, ‘Alternative antiretroviral therapy interventions’, ‘Group-based adherence intervention to antiretroviral therapy’, ‘Qualitative evaluation of group-based models’ and ‘in Sub-Saharan Africa’. Figure 3 presents the search process.

#### Inclusion criteria

We defined the inclusion criteria for the study selection of the review using the SPIDER (Sample, Phenomenon of Interest, Design, Evaluation, Research type) mnemonics for qualitative reviews [47].

- *Sample*: Stable adult (18+ years) patients on ART
- *Phenomenon of Interest*: Retention in care and adherence to antiretroviral medication
- *Design*: Quantitative, qualitative and mixed-methods studies
- *Exposure*: Facility-based and community-based group-ART models in SSA
- *Research type*: Primary research articles on group-based ART models

#### Exclusion criteria

- All articles that did not deal with a group-based ART model of treatment and care such as home-based care models.
- Non-English papers
- Papers reporting non-primary research

We used the ReadCube® Reference Management system to screen and manage the retrieval of the studies. The search was conducted in February 2016 and updated in April 2017. The date limit set on the records to search was 2005–2016 because group-based ART management models only emerged in the early 2000s.

### Ethical considerations

This study is part of a larger project ‘A realist evaluation of the antiretroviral treatment adherence club programme in selected primary health care facilities in the metropolitan area of Western Cape Province, South Africa’ which has received ethics clearance from the Higher Degree’s committee of the University of the Western Cape [41]. In addition, we followed the relevant standards of utility, usefulness, feasibility, propriety, accuracy and accountability while conducting the review [48].

### Data analysis—analysing the papers for the narrative synthesis

Conducting a NS involves four distinct but interrelated steps [43]:

- Developing a theory of how the intervention works, why and for whom
- Developing a preliminary synthesis of findings of included studies
- Exploring relationships in the data
- Assessing the robustness of the synthesis

#### Step 1. Developing a theory of how the intervention works, why and for whom

**Fig. 4:**
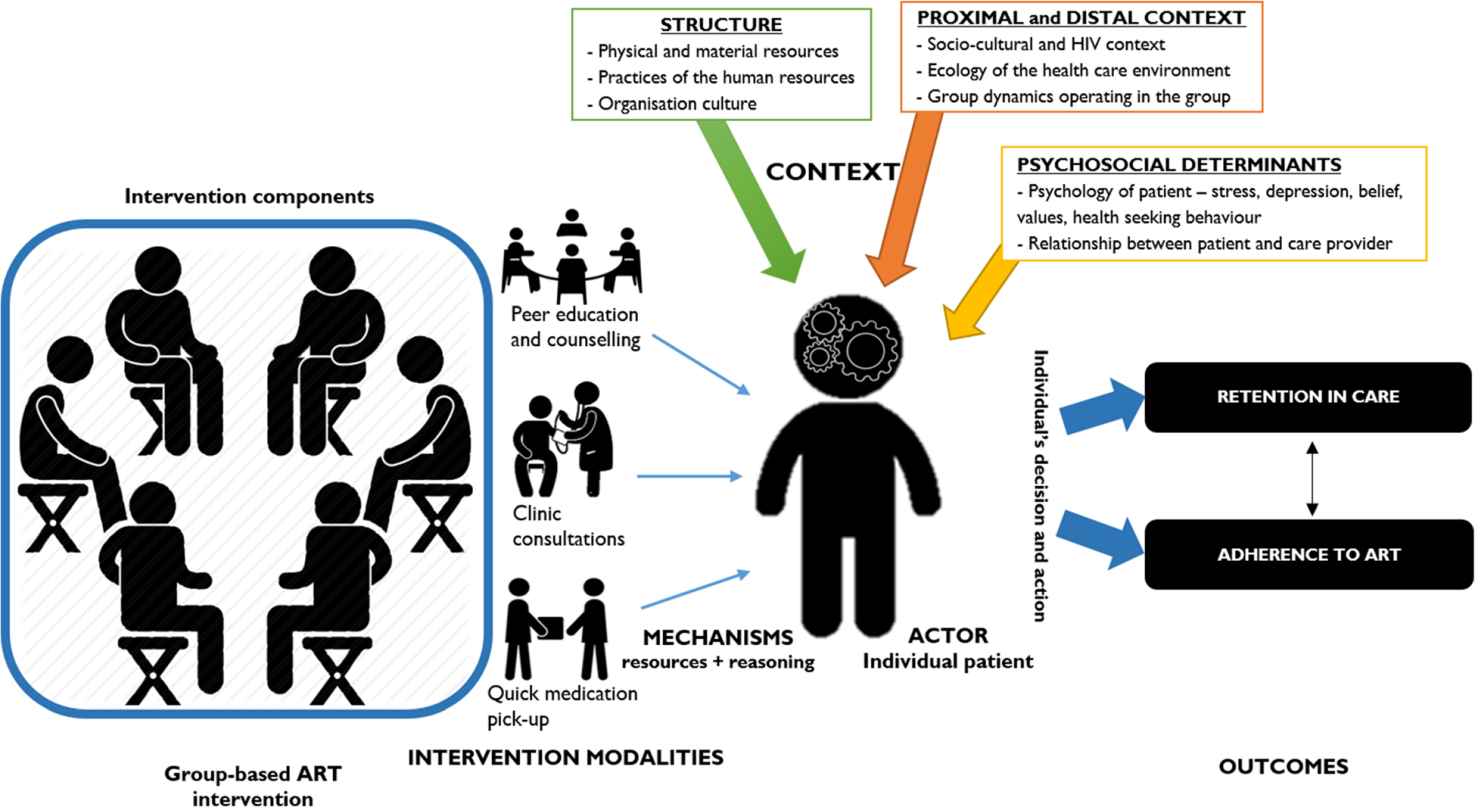
Representation of the initial theory of how group-based ART models work

We developed a theory on how the intervention under investigation works, why and for whom. This tentative theory is meant to guide the synthesis process and is to be refined at the end of the synthesis. We formulated an initial theory guiding this synthesis (Fig. 4) based on the principles of group-based ART models as outlined in the standards of practice of some of these group-based models [20, 49] and the folk theories that we formulated from the consultation with the adherence club programme designers and managers [50]. Group-based models of ART services have some basic principles guiding their implementation:

- Task-shifting of services to the lowest level of care provider, from nurses offering ART services to community health workers and in some instances to PLWHA (expert patients).
- Adherence is improved by decreasing the burden placed on patients (time, cost, pills) and by increasing the user-friendliness of care and treatment services.
- Having patients receive their care together to increase peer support among the patients and creating an enabling treatment and care environment.
- By separating the drug-delivery and clinical care and reducing the intensity of the services, the care process is simplified for the providers and made much user-friendly.

We associated the generic components of group-based ART intervention (intervention modalities, actors, context, mechanisms and outcomes) to formulate the initial theory (Fig. 4). The primary outcomes are adherence to medication and retention in care. The mechanisms were derived from underlying determinants or social behaviours identified in the folk theories and included motivation, trust, encouragement, social support, self-efficacy and buy-in. These mechanisms are expected to be triggered by the various programme components within the immediate (*micro*), organisational (*meso*) and distal contexts (*macro*) [51]. The contextual factors include human resources (staffing dynamics), stakeholder collaboration, availability of conducive physical space and support from the higher level of the organisation. The dashed lines in Fig. 4 indicate that the context goes beyond the immediate environment where the intervention is being implemented, including the organisational and the distal context.

### Data analysis/synthesis

#### Step 2. Developing a preliminary synthesis—extracting data from the included studies

In the second step, we extracted the data according to the following categories were used: (1) publication citation, (2) study country, (3) participants and setting where the study was conducted, (4) study design, (5) implementation setting and (6) description of the outcome (Table 2).

**Table 2:** Summary of the studies reviewed

Study	Intervention type: country	Description of sample sample size	Study design	Detailed description of outcomes
Decroo et al. (2011) [53]	Community ART group—alternative ART collection by a group member in Tete Mozambique.	Stable patients on ART (February 2008–May 2010) *N* = 1384	Cohort study	1269 (97.5%) were retained in care, 83 (6%) were transferred out, 30 (2%) had died, and 2 (0.2%) were lost to follow-up.
Decroo et al. (2014) [52]	Community ART group—alternative ART collection by a group member in Tete Mozambique.	Stable patients on ART (February 2008–December 2012) *N* = 5729	Retrospective cohort	Mortality and LTFU rates among 5729 CAG members were, respectively, 2.1 and 0.1 per 100 person-years. Retention was 97.7% at 12 months, 96.0% at 24 months, 93.4% at 36 months and 91.8% at 48 months.
Dudhai & Kagee (2015)[58]	Facility-based adherence clubs—Cape Town, South Africa	Adult ‘stable’ patients are forming groups of 15–30. *N* = 13 6 patients, 7 Health care workers	Descriptive qualitative design	1) The adherence club reduces the time ART users spent at the clinic. 2) Logistical problems associated with the timely and correct delivery of drugs. 3) Sense of belonging and cohesion among club patients 4) Patients become active participants in care rather than passive receivers of health care The adherence club helps to decongest the facility
Grimsrud et al. (2015) [30]	Community-based adherence clubs—Cape Town, South Africa	Stable patients are forming groups of 25–30. Down referred to an adherence club from May 2012–December 2013. *N* = 2133	Observational cohort	Over an 18-month period, 2113 patients were decentralised to one of 74 CACs (decongestion). LTFU among CAC patients was 2.6%, 3.9% and 6.2% at months 6, 9 and 12, respectively. Kaplan-Meier estimates of viral rebound were 1.4% at 6 months and1.7% at 12 months. Overall retention on ART was 97.2% at 6 months and 93.5% at 12 months.
Khabala et al. (2015) [60]	Medication Adherence Club—Nairobi, Kenya	Mixed groups of 25–35 stable hypertension, diabetes mellitus and HIV patients. August 2013–August 2014. *N* = 1432	Retrospective descriptive study	From a total of 2208 consultations, for both HIV and hypertension/diabetes patients, adherence appears to be high with blood pressure checked in 99%, weight checked by 98% and blood tests ordered correctly in 98–99% of patients. 2208 consultations, 43 (2%) were referred to the regular clinic. The overall loss to follow-up was 3.5% (30).
Luque-Fernandez et al. (2013) [29]	Facility-based adherence clubs—Cape Town, South Africa	Adult ‘stable’ patients are forming groups of 15–30. November 2007–February 2011. *N* = 502	Retrospective observational cohort	97% of Club patients remained in care compared with 85% of other patients. Club participation reduced loss-to-care by 57% and a viral rebound in patients who were initially suppressed by 67%.
Rasschaert et al. 2014 [27]	Community ART group—alternative ART collection by a group member in Tete Mozambique.	October 2011–May 2012 CAG patients and Stakeholders. 16 FGDs and 24 IDIs	Grounded theory	The CAG model provides cost and time savings for the patients, the certainty of ART access and mutual peer support resulting in better adherence to treatment. Patients also take more active role in their health care (self-management). Group members combine, share and develop their knowledge, experience and personal skills. At the community level, it has strengthened community action, empowered patients.
Rasschaert et al. (2014) [27]	Community ART group—alternative ART collection by a group member in Tete, Mozambique.	October 2011–May 2012 CAG patients and Stakeholders. 16 FGDs and 24 IDIs	Exploratory qualitative	(1) The CAG model was designed to overcome patients’ barriers to ART and was built on a concept of self-management and patient empowerment to reach effective results. (2) The daily management of the model is still strongly dependent on external resources, especially the need for a regulatory cadre to form and monitor the groups. (3) The model is strongly embedded in the community, with patients taking a more active role in their healthcare and that of their peers. (4) There is a growing enabling environment with political will and general acceptance to support the CAG model. (5) Contextual factors, such as poverty, illiteracy and the weak health system, influence the community-based model and need to be addressed.
Rasschaert et al. (2014) [27]	Community ART group (CAG)—alternative ART collection by a group member in Tete, Mozambique.	October 2011–May 2012 CAG Stakeholders. Quant data: February 2008–December 2012 Qualitative data: 16 FGDs and 24 IDIs *N* = 105	Mixed-methods design	The counsellors were considered key to form and monitor the groups. The main modifications found were the progressive adaptations of the daily CAG functioning and the eligibility criteria according to the patients’ needs. The CAG leads to cost and time-saving benefits and improved treatment outcomes. The model offered a mutual adherence support and protective environment to the members. The active patient involvement in several health activities in the clinics and the community resulted in a better HIV awareness, decreased stigma, improved health seeking behaviour and better quality of care.
Rich et al. (2012) [57]	Community-based ART treatment. Group enrolment and patient support group in Rwanda.	HIV-positive adults starting community-based ART treatment between June 2005–April 2006 *N* = 1041	Retrospective medical record review.	Among 1041 patients who initiated community-based ART, 961 (92.3%) were retained in care, 52 (5%) died and 28 (2.7%) were lost to follow-up. Median CD4 T-cell count increase was 336 cells/mL from median190 cells/mL at initiation.
Vandendyck et al. (2015) [56]	Community ART group (CAG)—alternative ART collection by a group member in Lesotho	Six- Eight Stable patients on ART January 2007 December 2010 Qualitative Sample: 8 FGDs and 40 IDIs *N* = 67 Quantitative Sample: *N* = 199	Mixed-methods design	One-year retention of among patients in CAG 98.7% and those not in CAG, 90.2%. The CAG members commented that their CAG membership 1) Reduced time, effort, and money spent to get a monthly ART refill. 2) Induce peer support, which enhanced adherence, socio-economic support and empowered members to deal with stigma; and 3) Resulted in the feeling of relief and comfort. 4) Village health workers confirmed increase openness about HIV in their community 5) Clinicians reported a workload reduction 6) Community-led indicated that CAG members promoted health seeking behaviour to the community members.
Venables et al. (2016) [56]	Medication Adherence Club—Nairobi, Kenya	*N* = 106 10 FGDs 19 IDIs with HIV-positive patients and patients with NCDs 15 sessions of observations	Qualitative design	1) MACs reduce stigma for HIV-positive patients 2) MACs reduce waiting times and prevented unnecessary queues

*FGD* focus group discussion, *IDI* in-depth interview, *N* sample size, *LTFU* lost to follow-up

Twelve articles representing six studies were identified. Seven of the studies were on the community ART group (CAG) intervention. Five of the studies’ settings were Tete, Mozambique [27, 52–55], one was in Lesotho [56] and the other one was in Rwanda [57]. Three of the studies examined an ART adherence club intervention implemented in South Africa [29, 30, 58]. Two studies in Kenya focused on the Medication Adherence Club implemented in Nairobi, Kenya [59, 60].

### Results

#### Step 3. Exploring relationships in the data and between studies: a realist perspective

**Fig. 5:**
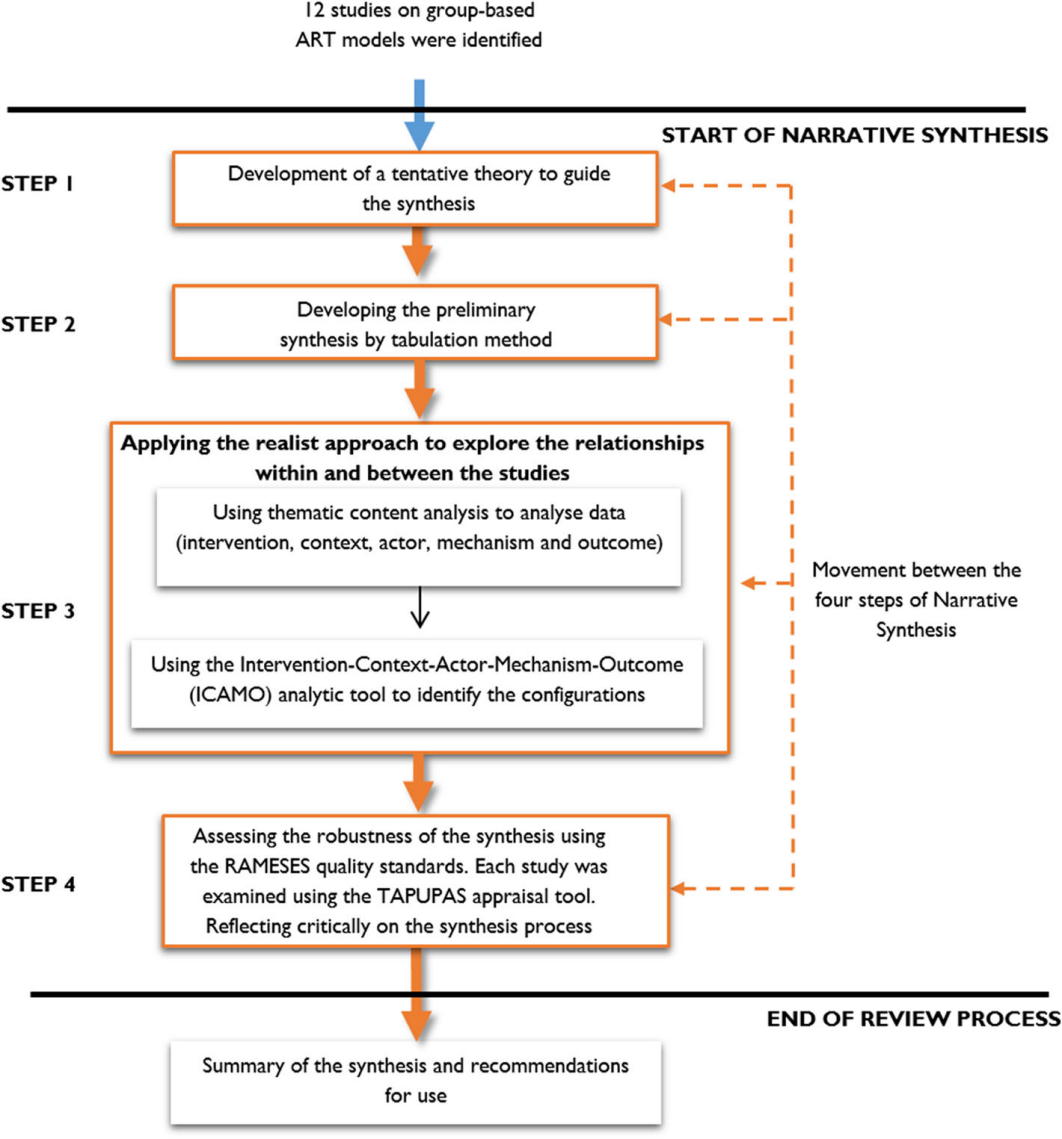
The analytical process of this narrative synthesis

We applied a two-phase process guided by realist evaluation principles (represented in step three in the synthesis process in Fig. 5).

First, we applied the thematic content analysis approach to systematically identify recurrent or salient themes across the selected studies. This was guided by core elements of the initial programme theory (see Additional file 1 for coding framework) (Table 3).

**Table 3:** Identification of recurrent or salient themes across the selected studies based on the realist logic

Study	Intervention modalities	Actors	Context	Mechanism	Outcome
Decroo et al. (2011) [53]	- A group representative visits the nearest health facility to collect medicines for the group. - Group members could still visit the health centre at any other time - A group meeting is held in the community before each clinic visit, and the designated group leader counts each members’ pills - The group representative meets with a clinician who prescribes ART and prophylactic drugs for each group member.	- Stable patients on ART - Adherence counsellor or clinician	- Poverty among ART patients - Perceived stigmatisation of patients when theyattend clinics - Treatments guidelines allow for one clinical consultation every 6 months and monthly supplies of medication.	- Building and reinforcing social networks and peer support - Encouraging greater patient responsibility	- Decrease the financial and economic/social costs of their treatment - Greater responsibility for the management of their own health
Decroo et al. (2014) [52]	- Community ART groups (CAG) - Peer support groups involved in community ART distribution - Mutual psychosocial support	- Stable patients on ART - Group of CAG members	- Difference psycho-social and biomedical characteristics than patients - Difference in adherence profile of patients in the CAG model	None identified^a^	- Mortality and loss to follow-up rates were better for patients in the CAG group than the clinic cohort - Retention in care rates with time was also improved.
Dudhai & Kagee [50]	- Facility-based antiretroviral adherence club	- Stable patients on ART	- Consistent and timely delivery of medication (failure) - Management of logistics by the host facility - Communication challenges between the host facility and the Chronic Dispensing Unit Staffing dynamics - need for more staff to run more clubs	- Cohesion among club members - ART users view themselves as active rather than passive participants in their care.	- Decongest the clinics so we have more time to spend with the sick patients or the new patients. - Shorter waiting time - Avoids financial loss on the part of the patient
Grimsrud et al. (2015) [30]	- Community-based antiretroviral adherence club intervention - Support ART maintenance for groups of stable patients in a community health worker-facilitated model with peer-support and increased patient self-management - Shifting the service away from health facilities to be community-based - Most CACs met five times per year	- Stable ART patients - Groups of 25–30 - Community health worker - A professional nurse was assigned as the CAC nurse rotating on a monthly basis.	- Limited resources within the community venue and distance to CHC for supplies - Policies regarding dispensing and distribution - Ensuring access to a clean and appropriate community-based facility - Limited resources within the community venue and distance to CHC for supplies	None identified^a^	- Better retention in care - Fewer people lost to follow-up and less attrition from the care programme
Khabala et al. (2015) [60]	- Medication Adherence Clubs - MACs are nurse-facilitated groups of 25–35 stable hypertension, diabetes mellitus and HIV patients who meet quarterly to (i) confirm their clinical stability, (ii) have a short health talk and (iii) receive pre-packed medications. - Routine patient follow-up with clinical officers occurs yearly when a patient develops complications or no longer meets the inclusion criteria.	- HIV and non-communicable disease patients - Professional nurse	None identified^a^	- Patient satisfaction	- An efficacious method of reducing clinicians’ workload - It also demonstrates a low loss to follow-up
Luque-Fernandez et al. (2013) [29]	Facility-based antiretroviral adherence club - Facilitated by non-clinical staff (counsellors) - Groups of 15 to 30 patients are formed and convene at the clinic during quiet times - Medicines are pre-packaged for each participant and brought to the group by a counsellor who weighs the patients and administers a symptom-based general health assessment. - Any patients reporting symptoms suggestive of illness, adverse drug effects or who have weight loss are referred to the clinic to be assessed by a nurse. - The counsellor or experienced patients lead short group discussions on health issues - A draw blood for viral load and CD4 count testing.	- Stable patients on ART - Non-clinical staff (counsellors) - Professional nurse	None identified^a^	- Group dynamic itself may be an important contributor as was historically motivated	- Administrative efficiency and decongestion of services are key aspects of the model - Improved retention in care might result due to the removal of these and other structural barriers to care - Virologic rebound was lower in the club model
Rasschaert et al. (2014a) [27]	- Community ART groups (CAG) - Based on the principles of self-management. - Patients rotate to pick up medication supplies for the rest of the group on a monthly basis - Each group elects a group leader, who functions as a spokesperson for the group. - The group members meet regularly in the community, perform monthly pill counts and offer mutual adherence support. - Lay counsellors, assist in forming and monitoring the groups in health facilities and the community	- Stable patients on ART - Group of CAG members - Involvement of other organisations likes MSF - Involvement of the Ministry of Health - Lay counsellors	- Progressive ministry of health involvement and integration of activities in existing health services - Flexibility to adapt to changing patients’ needs over time - Community participation - CAG model is well accepted by all stakeholders - Changed mindset of all stakeholders concerning the new health care approach - Continuous supervision, training and coaching sessions for patients and health staff - Low educational levels of most patients - Chronic shortage of staff	- Self-management and patient empowerment - Mutual adherence support - Increased assurance of timely access to ART - Motivation of care staff - Strong social links and networks between members	- Decreased workload and better monitoring of patients - Better general well-being - Less loss to follow-up and deaths - Improved adherence to treatment - Increased HIV awareness - Increased uptake of HIV testing, and a reduction of stigma
Rasschaert et al. (2014b) [54]	- Counsellor key role in forming and monitoring groups - GAC members participate in HIV-related activities in clinics and community - Group established CAG entry requirements - Flexible application of medical CAG eligibility criteria	- MSF employed counsellors - Stable patients on ART - Group of CAG members	- Permanent presence of counsellors in clinics - Resources for training and meetings - Consistent drug supply - Buy-in from the Ministry of Health - Problems with group formation, rotation system and relationships in groups	- Empowerment of patients - Mutual adherence support - Social control through ‘Code of Conduct.’ - Bonding between CAG members - Trust relationship - Patients are actively involved in their health decision-making - Problem-solving skills	- Better HIV awareness - Improved quality of care provided as supervision is in place - Decreased stigma - Improvement in the quality of health for patients - Better access to drug refills contributed to improved retention on ART.
Rasschaert et al. (2014c) [55]	- Groups comprise up to six stable patients on ART - Monthly, a group member is appointed to collect the drugs on behalf of the group and reports on and receives medical consultations for the group members. - Counsellors, sensitise patients to join groups and monitor the group activities.	- Stable patients on ART - Group of CAG members - MSF employed counsellors	- Weak healthcare system - Shortage in health staff - Lack of infrastructure - Discrimination and social exclusion when monthly attending the clinic. - Cultural beliefs that HIV is caused by spiritual spells and can only be managed by traditional healers - CAG intervention widely accepted among stakeholders	- Patients’ active role in health care - Social control and group rules - Psycho-social support - Understand the importance of taking medication - Very strong bond and network between the members.	- Reduced workload and improved quality of care in clinics - Better health outcomes - New identity of CAG members in group, clinic and community - The less frequent clinic visits per individual patient reduce the time and cost investment significantly - Better adherence to medication
Rich et al. (2012) [57]	- Patients qualifying for ART were given the option of entering a group of 12–24 persons for ongoing patient education and support. - Group enrollment consisted of a 3-h educational session and four individual visits before the initiation of ART. - After ART initiation, groups would attend routinely scheduled visits on the same day and meet for ongoing patient education and social support. - Routine visits occurred monthly for the first 10 months and then bi-monthly afterwards	- Patients qualifying for ART - Trained community health workers, also known as an “accompagnateurs,”	- Targeted support provided to health centres to ensure adequate staffing and retention of trained nurses, plus weekly physician supervision visits. - Trained CHWs, also known as an “accompagnateurs,” performed daily home visits. - Each patient received a monthly food package valued at the US $30 - Housing assistance, employment training and school fee support for patients and families in grave socioeconomic circumstances.	None identified^a^	- Good retention in care rates is retaining people in care at 2 years with very low rates of loss to follow-up and death.
Vandendyck et al. (2015) [56]	Community adherence group - PLWHA stable on ART was invited to constitute a CAG - CAG members meet monthly in the community. - During the meeting, they verify each other’s pill count (adherence) and choose a representative to go to the health facility. - At the health facility, the group representative has a consultation on behalf of the rest of the group members. - Then the representative returns to the community to distribute ART to the fellow group members	- PLWHA stable on ART - Community health workers	- Support from the village head - Separation of monthly ART refills from clinical assessments - Need for a reliable drug supply system to ensure access to ART - Availability of appropriate number of community health workers and lay counsellors to support the formation, training and monitoring of CAGs - Need for clear mechanisms to trigger support or referral back to clinic care to ensure patients and groups in need receive appropriate care - Availability of a simplified monitoring system to avoid increased administrative workload	- Being together, living in the same situation, bring the CAG to form a network of peers - Patients were empowered to take responsibility and to support each other. - Induced peer support, which enhanced adherence - Socio-economic support and empowered members to deal with stigma - Feeling of relief and comfort - Empowerment resulted from a new role for patients	- Village health workers confirmed increased openness about HIV in their community - Community leaders added that CAG members promoted health-seeking behaviour to community members - Clinicians reported a workload reduction. - Better retention in care within the first year of CAG membership. - Reduced time, effort and money spent to get a monthly ART refill
Venables et al. (2016) [59]	- Medication Adherence Clubs provide a medication refill system for stable HIV, diabetes and hypertensive patients. - Medications are pre-packed and labelled by the pharmacy - MACs are made of 10–30 stable hypertension, diabetes mellitus and HIV patients who meet quarterly to (i) confirm their clinical stability, (ii) have a short health talk and (iii) receive pre-packed medications. - Fast-track appointments - Routine patient follow-up with clinical officers occurs yearly when a patient develops complications or no longer meets the inclusion criteria.	- Stable HIV, diabetes and hypertensive patients - Non-medical health educators	- High prevalence of HIV, diabetes and hypertension - Support from a non-government organisation - Population living in informal settlements	- Patient satisfaction - Social support (mutual adherence support) - Acceptability related to advantages, - Empowerment	- MACs reduce waiting times and prevented unnecessary queues - MACs reduce stigma for HIV-positive patients

*MAC* Medication Adherence Club, *CAG* community ART groups, *CHW* community health worker

^a^No phrase corresponded to the definition of a mechanism as outlined in the coding framework

Following the thematic analysis, we explored the theoretical propositions assumed to represent the functioning of group-based ART models within each paper. First, we searched for the components of intervention, actors, context, mechanism and outcome within each article as they describe the group-based intervention in question. We found that the studies included for the review adequately described the intervention(s) under evaluation and their modalities. Similarly, the actors in the various studies were clearly identified in terms of the patient profiles, also the outcomes were well identified. Concerning the context, however, we observed that some studies provide more information than others but generally, there was a minimal structured discussion. Finally, virtually no paper presented a clear discussion on the generative mechanisms underlying the intervention under investigation. Indeed, we observed that all studies that only applied a quantitative research approach either failed to identify possible mechanisms or only identified a few [29, 30, 52, 53, 57, 60]. Very few studies identified some contextual factors around the implementation of group-based adherence models [54, 59, 60].

Because the studies that applied a quantitative design provided little or no information on the possible mechanisms and context conditions that explain the outcomes of the intervention, they provide little information that could help to theorise how group-based interventions work. The qualitative studies [27, 55, 58, 59] and the mix-method studies [54, 56] on the other hand, offered more information on the context, mechanism(s) and outcomes. Nevertheless, most of the information from the qualitative studies were produced by three studies conducted by the same group of authors evaluating a singular intervention (community adherence group) from different perspectives and another group of authors evaluating the Medication Adherence Clubs. These qualitative studies identified potential mechanisms and aspects of the context that could help explain the outcomes of the group-based models. Mechanisms identified from the selected studies include [perceived] social support (mutual adherence support), acceptability related to perceived advantages, empowerment, patient satisfaction, bonding among group members (trusting relationship), motivation, and increased assurance. However, none of the studies explored or demonstrated a relational association between the intervention, context, mechanism and outcomes nor did the studies show a causal explanation of how, why, for whom and under what circumstances do the interventions under investigation work (or not).

In summary, different studies identified different components of the ICAMO heuristic-analytic tool. Most of the studies did not identify all the components of the ICAMO heuristic tool. This was especially common with the studies that adopted the quantitative study approach. While some of the qualitative and mixed-methods studies identified most or all of the components of the ICAMO heuristic tool, none of the studies provided a conceptualised causal link of these components to explain how and why the interventions work in the context in which they are implemented.

### Rigour and trustworthiness

#### Step 4. Assessing the robustness of the synthesis

The inclusion of articles for the review process was based on two criteria: relevance and rigour. The RAMESES quality standards guided the quality assessment of the articles with regard to relevance [29], while the rigour was judged using a tool designed by the Centre for Reviews and Dissemination for Systematic Reviews in the Social Sciences [54].

We employed the TAPUPAS appraisal tool developed by Pawson and colleagues [41] to judge the relevance of the studies included in the review. This tool has the merit of not restricting itself to validity but also including other pertinent issues related to rigour such as ethics and accessibility [61]. The seven questions in the TAPUPAS appraisal tool embody statements of good principles in research and its reporting. The attributes of the TAPUPAS appraisal tool include [48]:

▪ Transparency: is the process of knowledge generation open to outside scrutiny?

▪ Accuracy: are the claims made based on relevant and appropriate information?

▪ Purposive: are the methods used fit for purpose?

▪ Utility: are the knowledge claims appropriate to the needs of the reviewer?

▪ Propriety: has the research been conducted ethically and legally?

▪ Accessibility: is the research presented in a style that is accessible to the reviewer?

▪ Specificity: does the knowledge generated reach source-specific standards?

Overall, the studies that were included in the review met all the criteria stipulated in the TAPUPAS appraisal tool. The studies that were considered relevant for the review were then scrutinised for rigour: each study was assessed in terms of quality. Different evaluation tools were used for different studies based on the study designs. The screening was done using an adapted extraction/critical appraisal form to guide data extraction of studies in a systematic review of the health and social sciences [62]. (Additional file 2).

Based on this assessment process, we concluded that the findings obtained based on these studies could provide valuable and credible information on which sound conclusions could be drawn. In addition, the study is reported in accordance with the Preferred Reporting Items for Systematic Reviews and Meta-Analyses (PRISMA) guidelines (Additional file 3).

## Discussion

This study is part of a large research project that applies the realist evaluation approach to study whether, how and why the adherence club intervention for the management stable patients on ART works. In realist research, the first step is to develop the initial programme theory. To do this, we first elicited the opinions and assumptions of the programme designers and managers (also called the folk theories). Based on these assumptions, we formulated tentative programme theories of the adherence club programme. In this paper, we sought to explore if the tentative programme theories that we formulated are compatible with research evidence (both applied research and basic research). We, therefore, conducted a narrative synthesis to identify and examine publications on group-based adherence models, looking at how those programmes were designed (especially their programme theory) and their effectiveness. We then assessed whether and how the studies produced evidence that could inform and refine our folk theories.

We found, not entirely unexpectedly, that the quantitative studies we identified did not provide sufficient information on the context and mechanisms that come into play to trigger the outcome. Although the four qualitative studies and the two mixed-method studies we found identified and described some of these elements, the association between these components and the explanation this may provide to account for the outcomes were not explored nor demonstrated. In other words, none of the studies provided evidence regarding the potential interplay between the aspects of the intervention, relevant context, significant actors, generative mechanisms and the outcomes of interest (causal explanation). This was identified by examining each of the identified studies through the ICAMO analytic tool. Consequently, they do not inform in an explicit manner how, why, for whom and in what circumstances that the evaluated group-based interventions work (or not).

Granting that the kind of studies we identified are useful in that they aim at assessing effectiveness, it would benefit the scientific community, policy makers and programme implementers more if details on the context and mechanisms of interventions designed to change behaviours were provided and if the evaluators would identify the causal processes underlying the observed results. This recommendation is echoed by calls made by implementation scientists to increase the use of theory to build knowledge about what works, where, and why [63]. Van Belle et al. also proposed that theory-driven approaches such as the realist evaluation approach have the potential to demonstrate the complex interplay between the components of a programme (or an intervention), relevant context conditions, actors involved, causal mechanisms and expected outcomes [64].

### Strengths and limitations

Using the NS allowed us to preserve the integrity of the findings of the different types of studies that are reviewed, as the method does not seek to quantify findings that are narrative or qualitative in nature, nor does it attempt to describe the qualities present in numeric data [65]. Also, the use of categorical codes allowed for mediating between two forms of data which helped us to move from reported findings to higher levels of abstraction [65]. However, applying this method required us to rely on the descriptions of interventions supplied by the authors, and this could be a potential limitation of this approach.

Other limitations are related to the use of only two databases for the search of the studies for inclusion in the review. This introduces the possibility of missing out other studies that could be relevant to the review objective fitting the inclusion criteria. This potential loss was compensated to a certain extent by conducting snowballing of references along with the database search.

## Conclusion

The types of studies identified in this review present a wide range in their evaluation approaches. Most of the quantitative-based approaches, unsurprisingly, provide little if any information on context and mechanisms. The qualitative studies somehow described context and mechanisms but did not go beyond description and conjecture. Because these studies did not identify nor demonstrate causal relationships, they did not provide information to guide the development of the initial programme theories underlying the ART adherence club interventions. In light of this challenges, we suggest that a way forward towards understanding how the adherence club intervention and other group-based adherence models work is to review the literature of other disciplines for possible theories on adherence to ART and/or chronic medications.

## Additional files

Additional file 1: Table S1. Coding framework. (DOCX 13 kb)

Additional file 2: Table S2. Extraction/appraisal tool. (DOCX 16 kb)

Additional file 3: PRISMA 2009 Checklist. (DOC 64 kb)
