# Supplementary material for: An exploration of group-based HIV/AIDS treatment and care models in Sub-Saharan Africa using a realist evaluation (Intervention-Context-Actor-Mechanism-Outcome) heuristic tool: a systematic review
Source: Implement Sci. 2017 Aug 25;12:107. doi: 10.1186/s13012-017-0638-0 (PMC5574210; doi:10.1186/s13012-017-0638-0)
Supplement: Supplementary file 1 — Coding framework. (DOCX 13 kb) [file 13012_2017_638_MOESM1_ESM.docx]

| Category | | Definition | Coding Rules |
| --- | --- | --- | --- |
| Actors | | These are the individuals, groups, and institutions who play a role in the implementation and outcomes of an intervention | This was coded as the actions or actual practices of an individual, group or institution. |
| Context | | Context refers to salient conditions that are likely to enable or constrain the activation of programme mechanisms. | Components of both the physical and the social environment that favour or disfavour the expected outcomes |
| Mechanisms | | This refers to any underlying determinants or social behaviours generated in certain contexts | Any explanation or justification why a service or a resource was used by an actor to achieve an expected outcome, or considered as a constraint |
| Outcomes | Immediate outcome | Describes the immediate effect of the adherence club programme activities | Immediate outcome typically refers to changes in knowledge, skills or awareness, as these types of changes typically precede changes in behaviours or practices. |
|  | Intermediate outcome | Intermediate outcomes refer to behavioural changes that follow the immediate knowledge and awareness changes. | Codes here define a move from direct outcomes to intermediate outcomes, identified through the indirect impact of the activity and accountability of the programme. |
|  | Long-term outcome | Refer to change in the medium- and long-term, such as a patient’s health status, and impact on community and health system | The codes here represent the further indirect impact of the activity demonstrating the lesser accountability of the programme. |

Additional File 1: Data coding framework
