# Supplementary material for: An exploration of group-based HIV/AIDS treatment and care models in Sub-Saharan Africa using a realist evaluation (Intervention-Context-Actor-Mechanism-Outcome) heuristic tool: a systematic review
Source: Implement Sci. 2017 Aug 25;12:107. doi: 10.1186/s13012-017-0638-0 (PMC5574210; doi:10.1186/s13012-017-0638-0)
Supplement: Supplementary file 2 — Extraction/appraisal tool. (DOCX 16 kb) [file 13012_2017_638_MOESM2_ESM.docx]

**Additional File 2: Research Evidence Extraction/appraisal tool**

| **Analyse Phase** | | | | |  | | | |
| --- | --- | --- | --- | --- | --- | --- | --- | --- |
| Article Title: | | | | | | | | |
| Authors |  | | | | | | | |
| Journal |  | | | | | | | |
| Setting |  | | | | | | | |
| Sample Size |  | | | | | | | |
| Objective of study clearly stated | | | | |  | | | |
| Study methodology or methodologies used | | | | |  | | | |
| Inclusion of sufficient data to assess validity of conclusions | | | | |  | | | |
| Data source | | | | |  | | | |
| Size of achieved sample and the population from which the sample is drawn | | | | |  | | | |
| Methods of measurement (data collection) | | | | |  | | | |
| Effect on behaviour | | | | |  | | | |
| Study type | - Meta-analysis | | - Quasi-experimental | | - Non-experimental | | - Qualitative | - Meta-synthesis |
| Does this study apply to the population targeted the review question? | | | | | | | - Yes | - No |
| **Strength of the study design** | | | | | | | | |
| Is the sample size adequate and appropriate? | | | | | | | - Yes | - No |
| Are the study participants randomised? | | | | | | | - Yes | - No |
| Is there an intervention? | | | | | | | - Yes | - No |
| Is there a control group? | | | | | | | - Yes | - No |
| If there is more than one group, are the groups equally treated except for the intervention? | | | | | | | - Yes | - No |
| Is there an adequate description of the data collection methods? | | | | | | | - Yes | - No |
| **Study Results** | | | | | | | | |
| Are the results clearly presented? | | | | | | | - Yes | - No |
| Is the interpretation/analysis provided | | | | | | | - Yes | - No |
| **Study Conclusions** | | | | | | | | |
| Are the conclusions based on clearly presented results? | | | | | | | - Yes | - No |
| Are the study limitations identified and discussed? | | | | | | | - Yes | - No |
| Pertinent study findings and recommendations | | | | | | | | |
| Will the results answer the review questions? | | | | | | | - Yes | - No |
| **Evidence Rating** | | | | | | | | |
| Strength of Evidence | | - Level I | | - Level II | | - Level III | - Level IV | - Level V |
| Quality of Evidence (Check one) | | | | | | - High (A) | - Good | - Low (c) |

Extraction form to guide data extraction of studies in a systematic review of the health and social impacts

Egan, M., Petticrew, M., Hamilton, V., and Ogilvie, D. Health impacts of new roads: A systematic review. American Journal of Public Health 2003, 93(9): 1463–71
